# A New Approach for Structural Monitoring of Large Dams with a Three-Dimensional Laser Scanner

**DOI:** 10.3390/s8095866

**Published:** 2008-09-24

**Authors:** Diego González-Aguilera, Javier Gómez-Lahoz, José Sánchez

**Affiliations:** 1 Cartographic and Land Engineering Department. University of Salamanca, Hornos Caleros, 50, 05003 Avila, Spain; E-Mail: fotod@usal.es (J. G-L.); 2 ECONTOP S.L. Avila, Spain; E-Mail: asanchez@econtop.es (J. S.)

**Keywords:** Active Sensor, Laserscanning, Point-based Techniques, Computer Methods, Structural Monitoring

## Abstract

Driven by progress in sensor technology, computer methods and data processing capabilities, 3D laser scanning has found a wide range of new application fields in recent years. Particularly, monitoring the static and dynamic behaviour of large dams has always been a topic of great importance, due to the impact these structures have on the whole landscape where they are built. The main goal of this paper is to show the relevance and novelty of the laserscanning methodology developed, which incorporates different statistical and modelling approaches not considered until now. As a result, the methods proposed in this paper have provided the measurement and monitoring of the large “Las Cogotas” dam (Avila, Spain).

## 1. Introduction

Monitoring the static and dynamic behaviour of large dams has been always a topic of great relevance, due to the impact that these structures have on the landscape where they have been built. The actual responses of dams may differ from the initial values computed at the design state for several reasons. These are: the type of construction, assumptions made in structural modelling and analysis, differences between the proposed design and the built structure, the unpredictable and changing nature of loads. Therefore, constant monitoring of the structural response is needed to secure and preserve the safety of this type of structures. In fact, this field has received a growing attention in the last few years through the activities of FIG (International Federation of Surveyors) Commission 6, since they have an active research agenda in this area [7]. Many instruments and surveying methods have been used in order to support the control of these structures. However, the main aim in most of the developed plans has been to ensure the possibility of measuring displacements in a singular number of points. The difficulty in the measurement of these displacements is to find a spatial measurement technique that encompasses numerous desirable properties, such as, precision, reliability, low cost and easiness to use. Some of these advantages can be seen in several methods, but it is really hard to find a method comprising all of them. In the next paragraphs some of the approaches developed in this context are reported (starting with the classical techniques and finishing with the new technologies):

- *Classical topographic methods* based on angles, distances and height variation measurementsare very popular in the quantitative surveying field. The equipment used consists of accurateand appropriate theodolites or total stations. When the point that has to be determined isinaccessible, indirect methods are used, for example: single or multiple intersections [9].Furthermore, contact sensors can complete these measurements, such as: an inclinometer, apendulum, dial gauges or extensometers. However, this contact nature prevents them from useat the final stages of destructive load testing, and they can only acquire measurements in onedimension.
- *The Global Positioning System (GPS)* has been used in structural monitoring of large dams [13, 17], [19-21], as well as combined with other sensors such as accelerometers [14] and inertialnavigation systems [12]. In spite of this, GPS has two significant limitations, as well. Firstly, assignals are received from satellites, coordinates cannot be measured indoors or throughobstacles. The second limitation is that the current precision levels of GPS are limited to +/- 1cm horizontally, and +/- 2cm vertically [3].
- *Digital close-range photogrammetry* has been a low cost alternative [2], and is highly accurate [8]. It also offers a quick, remote, three-dimensional data acquisition with images that provide apermanent visual recording of the test, but the compulsory use of targets might bedisadvantageous in some circumstances; especially when the access to the object is risky orwhen it is inaccessible to operators. Due to the lack of scale definition in the photogrammetricprocess, measurements must be taken by using additional instrumentation (for example, reflectorless total stations).
- *Terrestrial Laser Scanning* has become a new alternative to the monitoring of structuresincorporating novelty approaches [18] and computer methods [11]. Although the approachesnoted above present an accurate modelling strategy and have demonstrated their viability forstructural monitoring, none of them has been tested yet over complex structures such as largedams. In this context, [1] outlined some first results of a project aimed at monitoringdeformations of large concrete dams by terrestrial laser scanning. The reported analysis focuseson two main problems: the first one is the accuracy and the stability of georeferencing, which isfundamental to make comparisons between different multi-temporal scans; the second one isthe computation of deformation based on the acquired point-clouds. Particularly, a comparison is performed using different surfaces types, such as: resampled point cloud, mesh and polynomial surface.

The approach proposed in this paper deals with the application of Terrestrial Laser Scanner (TLS) to the structural monitoring of a large dam considering aspects related to the accuracy control in georeferencing, together with rigorous approaches to model complex structures. Particularly, the novelty and importance of this approach lays on the utilization of the Radial Basis Function (RBF) for the surface parameterisation, as well as the incorporation of an original re-Weighted Extended Orthogonal Procrustes (WEOP) analysis for georeferencing and the accuracy control of the different measurement periods.

The paper presents the following structure and organization: after this introduction, Section 2 describes in detail the methodology developed for deriving the structural monitoring. Section 3 outlines the results obtained. A final section is devoted to put across some analysis and conclusions.

## 2. Methodology

The methodology needed for the structural monitoring of the concrete “Las Cogotas” dam is divided into two stages. The first one is the field work. At this stage, the information is obtained using TLS following basic rules and protocols. The second stage is the laboratory work. At this stage, the information is processed in order to deliver results and to asses their significance and quality.

### 2.1. Field work

Field work can be summed up into the following stages: (a) Sensor selection; (b) Range data acquisition; and (c) Accuracy control.

#### Sensor selection

The periodical monitoring of the structure is performed with a TLS, the Trimble GX200 [22]. Based on the TOF (Time Of Flight) measuring principle, this scanning system allows to cover a large field of view (360° H × 60° V) thanks to the adoption of a beam deflection system based on an oscillating mirror. Furthermore, besides the intensity of reflected beam, the GX200 is able to acquire also RGB data, through the real-time video camera with a resolution of 768 × 576 pixels. The specifications of the TLS used in this study, including the diameter and interval of laser spots formed when individual laser pulses are reflected on an object, are summarized in Table 1 in terms of the distance to the structure in meter. The laser spot spacing increases linearly when the measurement distance increases.

#### Range data acquisition

The principle of 3D coordinate extraction using TLS is based on measuring the time it takes for the laser pulse to travel from its source to an object and return. The distance is computed from the travel speed of the pulse (the speed of the light). Relative 3D coordinates of an object with respect to the TLS are obtained by using the distance and laser pulse angle data generated from emitting laser pulses to the object. The three-dimensional range data are acquired along three different measurement periods. One single laser scanner station is used at a mean distance of 100 m from the main wall surface in each measurement period. In spite of this restriction, the TLS is not set up strictly over a known point, so the position and orientation of the TLS is determined by resection from a reference coordinate system defined through high precision topography. Additionally, taking into account that the GX200 incorporates an automatic dual axis compensator, the TLS is carefully leveled each time in order to define the vertical direction, providing a geometric constraint to check the vertical direction of the reference coordinate system. As the resection process will cause additional errors to arise, it is a practical task to enable direct comparisons of vertical directions from the topography and TLS data sources.

In relation to the range dataset resolution, those parts subject to major deformations (central portion of dam), are scanned with a resolution of 3 mm; while the rest of dam is scanned with a resolution of 2 cm. Moreover, control points in the reference coordinate system represented by artificial targets are automatically scanned with the highest TLS resolution, 3 mm. These targets are placed out of the object of study and fixed in stable elements such as control survey marks, in order to establish an external reference frame free of possible deformations. As a result, a total of 8.5 millions of points are obtained to model the complete structure. This data acquisition protocol has been carried out for each measurement period maintaining the same criterions strictly.

#### Accuracy control

**Figure 1.**
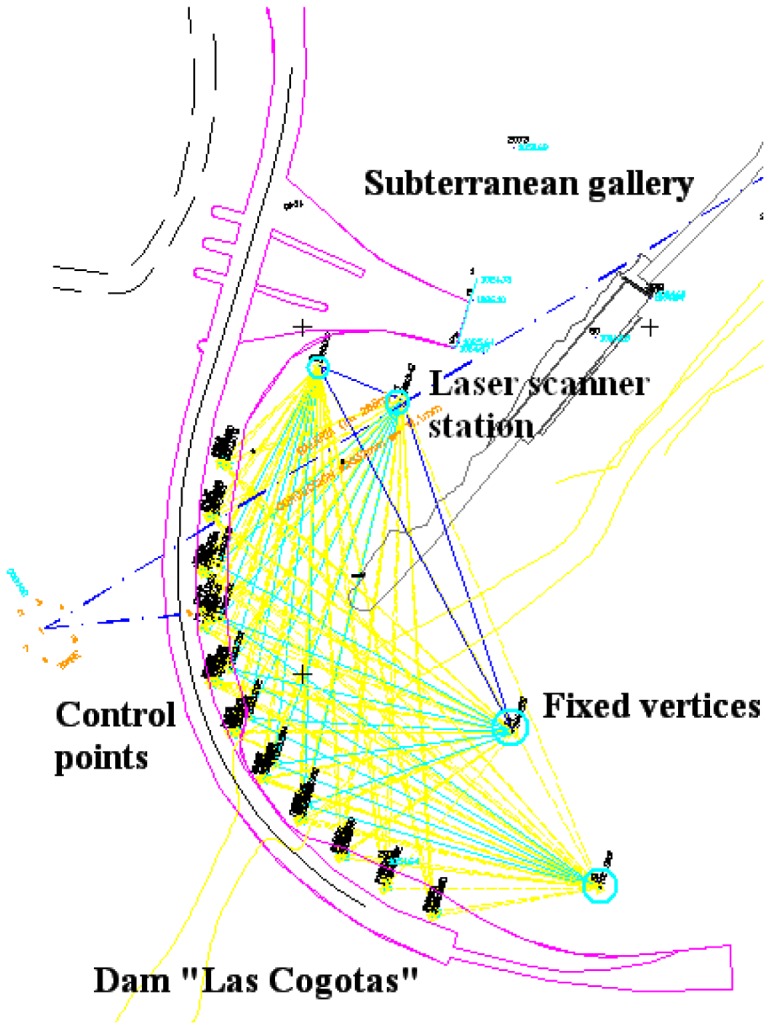
Accuracy control based on a network of fixed and stable control points.

In order both to provide an accuracy control of the point-based techniques and to guarantee a correct georeferencing of the multi-temporal periods, a high precision topographic equipment is utilized for establishing the reference coordinate system. Thus, high precision topographic techniques are used to benchmark the TLS measurement periods and to establish an accuracy control of the TLS georeferencing. Due to the complexity of the large dam, a network design composed by four stable and fixed vertices (control survey marks) and six more control points (targets) have been used to materialize the reference frame. Besides that, several control points situated in the main wall of the dam have been surveyed to check the surface parameterisation. These control points are measured based on multiple intersection techniques, taking angular and distances measurements (Figure 1). The horizontal angles are observed by directional method, reading the horizontal circle in both the backsight and foresight directions. Multiple observations of the angle are made, with the circle being advanced prior to each reading to compensate for the systematic errors. Each angle is measured six times (3 direct and 3 reverse). The final angle is taken as the average of all measured values. Likewise, a total of six distances (3 direct and 3 reverse) are observed for each control point. The final distance is taken as the mean of all measured values. A Leica TCA2003 total station is used. This instrument is motorized and allows angular and distance accuracies of 0.5 ” and 0.5 mm for distances below 120 m respectively. Other technical features are: laser plumb line; magnification 30x; electronic level sensibility; twofold axis compensator. More features are referred to vendor's documentation [15].

### 2.2. Laboratory work

Laboratory work is a subsequent stage to field work. Laboratory work serves the purpose of processing previously gathered information. It is carried out in three stages: the first one involves the range data modelling; the second georeferences both dataset and establishes an accuracy control frame; the third monitors and analyses the different TLS periods. To this end, several tools which incorporate Matlab libraries have been developed for testing the different experiments.

#### RBF surface modelling

With the aim of analyzing the health monitoring of the structure, the point cloud data must be interpolated and reconstructed as a 3D surface model. In this case, considering that the object of study (large dam) presents a complicated geometry with the presence of several defects, damages and deteriorations, the RBF surface modelling has been adapted as follows:

- Incorporating RBF for the surface parameterisation of a complex and large structure.
- Demonstrating that modelling unorganized range data sets and topologically complicated objects is possible.
- Introducing RBF centre reduction without loss in accuracy.
- Implementing this approach using tools developed by the authors.

In particular, our method involves two steps:

1. Constructing a signed-distance function.
2. Fitting RBF to the resulting distance function.

In the first step, a si gned-di stance function is constructed from the surface data by specifying off-surface points along surface normals. A function ***f*** which implicitly defines a surface *S'* and satisfies the Equation (1) should be found.

(1) $$\begin{array}{ll} f(x_{i},y_{i},z_{i})=0, & i=1,\ldots ,n. \end{array}$$

where {(*x_i_ y_i_ z_i_*) }^n^_i=1_ are points lying on the surface. In order to avoid the trivial solution that ***f*** is zero everywhere, off-surface points are appended to the input data and are given non-zero values. This leads to a more useful interpolation problem: Find ***f*** such that,

(2) $$\begin{array}{lll} f(x_{i},y_{i},z_{i})=0, & i=1,\ldots ,n. & (\text{on}–\text{surface points}),\\ f(x_{i},y_{i},z_{i})=d_{i}\neq 0, & i=n+1,\ldots ,\text{N} & (\text{off}–\text{surface points}), \end{array}$$

This still leaves the problem of generating the off-surface points { (*x_i_ y_i_ z_i_*)}*^N^_i=n+1_* and the corresponding values *d_i_*. An obvious choice for ***f*** is a signed-di stance function, where the *d_i_* is chosen to be the distance to the closest on-surface point. Points outside the object are assigned positive values, while points inside are assigned negative values. These off-surface points are generated by projecting along surface normals. In the present case, considering the unorganised point-cloud data, normals are estimated using additional information such as scanner position to resolve the sense of the normal. In the second step, given a set of zero-valued surface points and non-zero off-surface points, a fitting of an RBF to the resulting distance function is established (3), approximating the signed-di stance function ***f*** by an interpolant *s*(*x*),

(3) $$\begin{array}{ll} s(x_{i})=f_{i}, & i=1,\ldots ,n \end{array}$$

In this case, the RBF function uses a polyharmonic spline (4) to reconstruct the surface from point cloud data,

(4) $$s(x)=p(x)+\sum_{i=1}^{N}\lambda_{i}\Phi (x-x_{i})$$

where *s* is the RBF function, *p* is a quadratic degree polynomial, *λ_i_′_s_* are the RBF coefficients, *Φ* is a real value function called the “basic function”, and x*_i_′_s_* are the RBF centres.

In a specific manner, the basis function used in this case is a triharmonic spline *Φ* (*r*)= *r^3^* with a quadratic polynomial whose coefficients are 1,*x,x^2^,y,yx,y^2^,z,zx,zy,z^2^*. Likewise, RBF centre reduction has been taken into account.Conventionally, an RBF approximation uses all the input data points (the x_i_ in Equation (3)) as nodes of interpolation and as centres of the RBF. However, the same input data may be able to be approximated to the desired accuracy using significantly fewer centres. To this end, a simple algorithm has been used to iteratively fit an RBF to within the desired fitting accuracy, considering the following steps:

1. Choose a subset from the interpolation nodes x_i_ and fit an RBF only to these.
2. Evaluate the residual, *ε_i_* = *f_i_*-*s* (*x_i_* ), at all nodes.
3. If the maximum residual {|*ε_i_* | } is inferior to the fitting accuracy then stop.
4. Else append new centres where *ε_i_* is large.
5. Re-fit RBF and go to step 2.

Therefore, reducing the number of RBF centres results in smaller memory requirements and faster evaluation times, without a loss in accuracy. The results presented in Section 3 (see Table 5) show that dense laser scan data can be represented by significantly fewer centres than the total number of data points. Finally, the accuracy of the method is assessed by means of the maximum difference between the fitted RBF value and the given values (5),

(5) $$\max_{i=1,\ldots ,n}\left|s(x_{i})-f_{\text{i}}\right|$$

#### Georeferencing and accuracy control of range dataset

Considering that a fundamental step in health monitoring of structures is the reference coordinate system definition, two main aspects must be taken into account: (i) georeferencing must be accurate and reliable enough; (ii) the georeferencing accuracy must be assessed.

Procrustes analysis exhibits significant improvements compared to conventional least squares adjustment, especially advantages related to accuracy, computing cost and operator handling. However, the most important disadvantage is its lack of reliability in order to detect and localize gross errors, which might have been included in the dataset. Likewise, its lack of analysis capability of the stochastic model and the possible existence of correlation among all the measurements, can lead to completely wrong results and might even prevent convergence of the adjustment. So, the georeferencing reliability must be assessed.

With the aim of avoiding those limitations remarked above, the Weighted Extended Orthogonal Procrustes analysis (WEOP) has been modified incorporating a robust estimator. Particularly, an original georeferencing approach based on a re-weighted Procrustes analysis has been developed. The analysis consists of a least squares method for fitting a given matrix **A** to another given matrix **B** under choice of an unknown scale factor *c* in such way as to minimise the trace of **E^T^WE,** that is, tr (**E^T^WE**)=min, where E is computed as follows

(6) $$\text{E}=c\text{AR}+\text{j}{\text{t}}^{\text{T}}-\text{B}$$

where matrices **A** and **B** are (*n* × 3) corresponding point matrices, **R** is the (3 × 3) unknown orthogonal rotation matrix under its orthogonal condition that is (**R^T^R**=**I**), ***t*** is the (3 × 1 ) unknown translation vector, ***j*** is a (*n* ×) unit vector which describes the number of corresponding n points, *c* is the scale factor, **W** is the (*n* × *n*) weighting diagonal matrix of the ***n*** points and **E** is the (*n* × 3) residual matrix.

At the first attempt, we assume that **W** = **I**, then the weights are updated iteratively using a robust estimation function. The modified Danish robust estimator [6] is used considering the following expression (7)

(7) $$w_{D_{\text{i}}}\left({\varepsilon_{i}}^{\prime }\right)=e^{\left(-{\left|{\varepsilon^{\prime }}_{i}\right|}^{2}\right)}$$

where ***w****_Di_* represents the weight function and *ε_i_ ′* is the quadratic component of each row of the residual matrix **E**. The re-weighted Procrustes analysis with these new weights (***w_Di_***) is repeated. The iterative process continues until the convergence is achieved. The convergence criterion is established based on the variation of the trace of the matrix, **E^T^WE**, from iteration to iteration, so the iterative process stops when this variation is not significant.

The degree of adjustment between the matrices **A** and **B** is assessed by means of the root mean square error (RMSE) (8),

(8) $$\text{RMSE}=\sqrt{\frac{\text{tr}\left({\text{E}}^{\text{T}}\text{WE}\right)}{\left(\text{n}-1\right)}}$$

where **E** is the residual matrix, **W** is the re-weighted matrix based on modified Danish robust estimator. The closer to zero RMSE is, the better the adjustment is.

Besides RMSE, the accuracy of the method has been assessed by means of a factor that shows the differences in distance̵s between control points provided by the TLS and control points provided by topographic instruments (Leica TCA2003 total station). This accuracy of the method has been assessed according to the following expression (9):

(9) $$\begin{array}{ll} df=\sqrt{\sum_{i=1}^{N}\sum_{\text{j}=2}^{N}{\left[{\left({d^{j}}_{i}\;\right)}_{T}-{\left({d^{j}}_{i}\;\right)}_{L}\right]}^{2}/n,} & i<j \end{array}$$

where (*d^j^*_i_)*_T_* is the distance between control points i and j measured by means of topographic methods, and (*d^j^*_i_)*_L_* is the distance between control points i and j measured by means of the laser scanner. *df* values close to zero suggest that the relative location of the points according to both systems is very similar.

#### Structural monitoring

The final goal of this study is to evaluate the possibility of performing a deformation analysis by means of TLS data. Thanks to the TLS technique it is possible to acquire a dense and accurate point cloud describing the external surface of the dam. Unfortunately, even though different scans have been georeferenced into a stable and accurate reference system, the deformation analysis cannot be carried out by considering points directly. This is due to the impossibility of scanning the same point in different measurement periods, because of the imperfect repositioning of the instrument and because of the laser beam width [16].

In the present case, the structural monitoring developed method is based on testing whether deformation occurs between two periods of structure measurements or not. The use of a parametric 3D surface generation technique based on a RBF function has allowed us to overcome the single point accuracy of TLS, seeing that surface elements derived from a large number of points could originate deformation parameters. As a result, a surface analysis is performed comparing data acquired in different periods and georefering them into the same reference system. Particularly, a deformation analysis using multiple orthogonal cross sections (horizontal and vertical) is applied to 3D surfaces. With the resulting sections we can extract automatically 3D displacements vectors not only applied to a limited number of points, but also to the whole dam structure. As a result, a map of variations which includes the displacements between different measurement periods is obtained.

## 3. Experimental results: the “Las Cogotas” concrete dam

**Figure 2.**
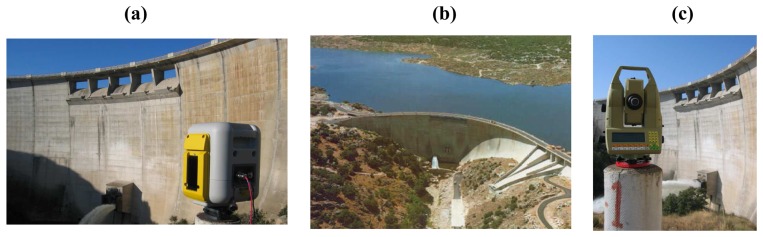
**(a)** Trimble GX200 laser scanner. **(b)** Downstream face of the dam of “Las Cogotas”. **(c)** Leica TCA2003 total station.

The ‘Las Cogotas’ concrete dam was built in 1994 on the Adaja river, generating a basin of about 58.6 Hm^3^ of water. The dam presents an arc structure featuring a height of 66 m and a length of 300 m at the crest ,with a maximum water level height of 1,050.50 m above sea level. The conservation of the structure is good, thanks to the monitoring which is carried out by traditional sensors (strain gauges, inclinometers, etc.) and by periodical geodetic measurements (leveling and geodetic control networks, optical collimators). The morphology and orography of the ground and its difficult accessibility, (Figure 2) makes scanning surveying specially interesting to monitorize the structure.

The following results have been useful not only for showing its viability, but also to secure and maintain the safety and serviceability of this structure. To evaluate the performance of the proposed methodology for structural monitoring, three different measurement periods were executed with different aims (Table 2). The first measurement period takes place in May 2006 when the dam is almost empty and its main goal is to provide an initial state (null deformation) of the structure. With this aim, a full and detailed metric digital archiving is obtained based on TLS. In addition, this measurement period claims to establish an independent assessment of the TLS periods through a network design based on high precision topography. Four stable and fixed vertices (control survey marks) together with six more control points (targets) are set up for the network design (Figure 1).

These targets are placed out of the object of study and fixed to stable elements such as control survey marks, in order to establish an external reference frame free of possible deformations. Afterwards, a network adjustment is performed based on least squares adjustment and a free network solution. As a result, a reference coordinate system (datum) was materialized through high precision topography. Table 3 displays the standard deviations resulting from the network adjustment. The final average standard deviation in the reference coordinate system is 2 mm in *X, Y* and 3 mm in *Z*.

Using the values of the high precision topographic network as a reference coordinate system and the TLS coordinates of the control points, a re-Weighted Extended Orthogonal Procrustes (WEOP) analysis is carried out. Table 4 displays the values of the parameters of transformation from TLS coordinate system to the topographic system using WEOP, and the root mean square error calculated according to Equation (8).

In particular, the degree of accuracy for the reference system definition, the RMSE, which measures the goodness-of-fit between both systems (laser scanning and topography), is 3 mm. A scale factor *c* close to 1 suggests that the distance between control points is similar according to both systems. Concretely, the scale factor between topographic (reference) and laser scanning (destination) represents a change in range of 1 cm, which can be considered acceptable taking into account the TLS single point accuracy according to the vendor's documentation [22]. Furthermore, the value of the parameter df, given by equation (9), is 2 mm, which is another argument in favour of the accuracy of the control points surveyed by laserscanning once the WEOP parameters have been incorporated. Likewise, this factor performs as a comparison parameter between the total station and TLS “control” dataset. Similarly, in order to assess the goodness of the automatic leveling compensation provided by the TLS, the following expression (10) has been computed using the displacements obtained in the WEOP analysis (Table 4), and calculating the quadratic differences between the laserscanning *Z* coordinates (*Z_L_*) and topographic *Z* coordinates (*Z_T_*), divided by the number of control points minus 1 (10):

(10) $${\text{Leveling}}_{\text{z}}\_\text{Effectiveness}\to \sqrt{\frac{\sum_{\text{i}=1}^{\text{n}}{\left(Z_{L}-Z_{T}\right)}^{2}}{n-1}}=0.002\text{m};\;n=10$$

Therefore, this equation measures the effectiveness of the leveling compensation by the tilt angles and not the leveling accuracy, since any accidental errors in the Z coordinates due to the TLS being off level are compensated by *ω* and *φ* angles of the transformation. This is the reason for which the estimated angles (Table 4) are so large. For example, the linear equivalent displacement of the *φ* angle is 6 cm at 100 m.

**Figure 3.**
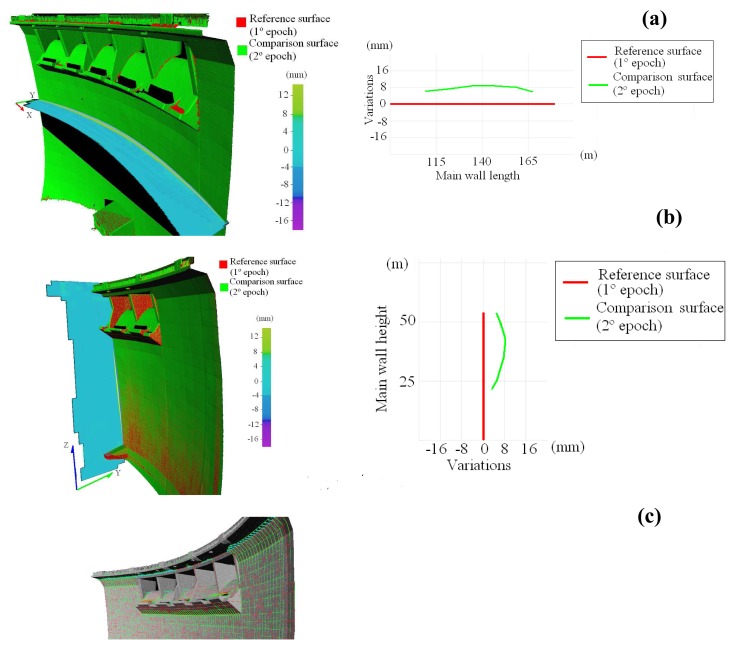

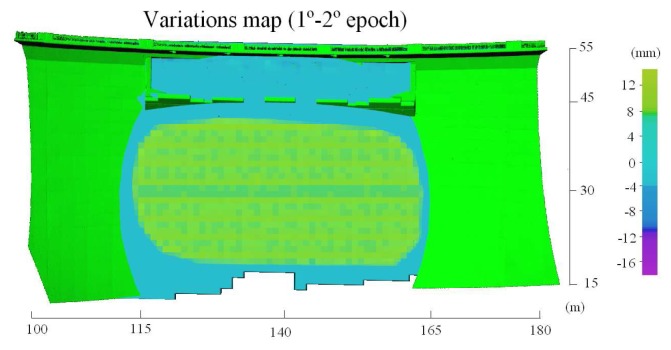
(**a**) Structural monitoring of the dam between 1^st^ and 2^nd^ measurement periods. Horizontal cross-section and variation scatterplot values. (**b**) Vertical cross-section and variation scatterplot values. (**c)** Multiple cross-sections applied over RBF 3D surfaces and variations map resulting of structural monitoring.

After six months (October, 2006) when the dam is completely full, a second measurement period is performed with the aim of confirming possible deformations of the structure. As such, an accurate and reliable deformation measurement must be provided in order to quantify the behaviour of the structure. After processing the range dataset corresponding to the first and second period (Table 5), a structural monitoring is established between both measurement periods based on multiple orthogonal cross sections applied over parametric 3D surfaces (Figure 3).

The RMSE obtained in the parametric 3D surface in both periods suggests that the RBF approach is well suited to reconstruct surfaces from non-uniformly sampled data, even on the basis of millions of points. Furthermore, 3D surface accuracy has been checked based on topography using several control points located in the main wall of the dam. This accuracy yields values around 1-2 mm which can be considered acceptable enough.

From the analysis of Figure 3 the following aspects can be asserted: the deformation derived from the structural monitoring between first and second measurement periods presents a value according to the expected structural behaviour of the dam. In particular, the dam experiments a movement downstream of 8 mm, due to its increase of capacity from May to October. Likewise, this deformation is higher in the centre of the main wall and decreases towards its extremes. This behaviour is compatible with the theoretical and periodical values expected for this dam.

**Figure 4.**
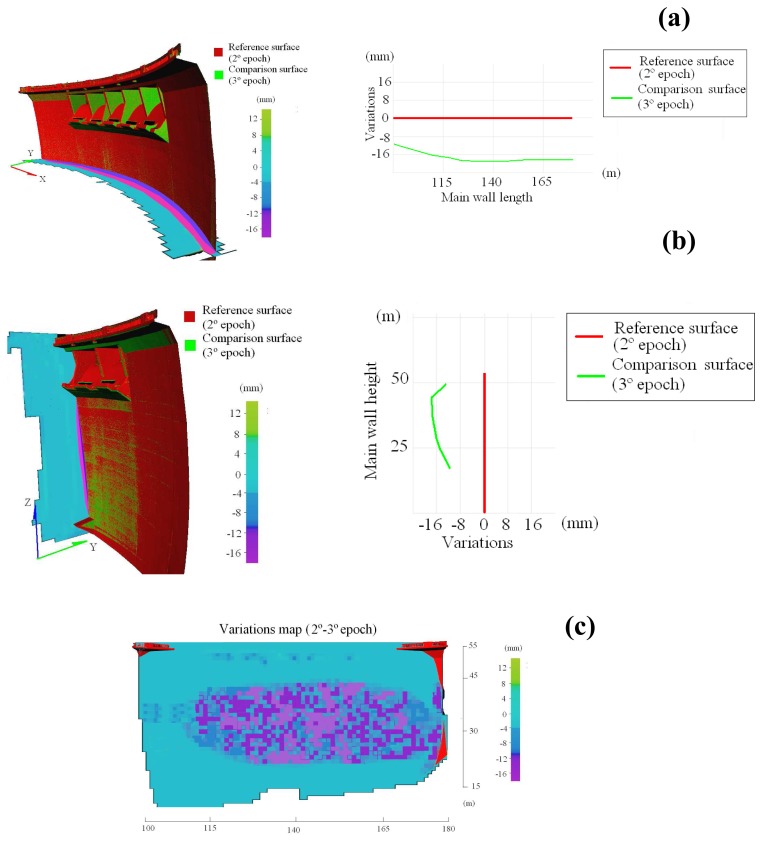
(**a**) Structural monitoring of the dam between 2^nd^ and 3^rd^ measurement periods. Horizontal cross-section and variation scatterplot values. (**b**) Vertical cross-section and variation scatterplot values. (**c**) Variations map resulting of structural monitoring.

After these two measurement periods an important civil engineering work, a subterranean gallery, was executed. Concretely, in order to exploit the dam as a hydroelectric resource, a subterranean gallery was built to connect the dam with a small electric central. This gallery has a length of 265 m with a circular section of 2.032 m. Due to its proximity to the dam, a new measurement period was performed in March 2007, to confirm its stability and check for possible strange movements due to the construction of the gallery. To this end, the range dataset corresponding to the third period are processed (Table 6) and a structural monitoring is established between the second and third measurement periods (Figure 4).

From the analysis of Figure 4 the following aspects can be asserted: the construction of the subterranean gallery has increased the displacements with relation to the expected values, that is, those corresponding to its decrease of capacity between the second and third measurement periods. This seems to be logical especially if we consider that several controlled blasting have taken place. In this sense, the deformation derived from the structural monitoring between second and third measurement periods presents a higher value according to the expected structural behaviour of the dam. In particular, the dam experiments a deformation upstream of -18 mm. Furthermore, this deformation does not decrease on the right lateral part of the dam due to the proximity of the subterranean gallery.

Finally, in order to confirm the results, a structural monitoring is established between the first and third measurement periods. In particular, the dam experiments a deformation upstream of -10 mm, mainly due to the construction of the subterranean gallery and also to its decrease of capacity. This value also allow us to confirm that the deformation effect of the 1th-2th and 2th-3th periods counteract each other and provide a low deformation. In addition, the deformation remains on the right lateral part of the dam which confirms the initial hypothesis.

## 4. Analysis of Results and Conclusions

The presented paper has investigated and tested the viability and possibilities offered by TLS in the control and monitoring of a large dam. A consistent and reliable full process has been developed and analyzed, from which the following considerations can be outlined: a TLS sensor alone is not enough for providing a structural control of large dams, due to the impossibility of scanning the same point in different measurement periods and the laser beam width. Nevertheless, their extraordinary capacity for acquiring data (points) massively makes them unsurpassable in comparison to whatever geomatic technology. Thus, this paper has been focused on two main points: provide a 3D modelling approach which improves the nominal precision of the TLS and establish a precise georeferencing of the different measurement periods. For the first goal, a surface 3D modelling based on RBF functions has allowed us to carry out an exhaustive structural monitoring applied to the large dam, especially for fitting surfaces to non-uniformly sampled point-clouds and partial meshes that contain large irregular holes. In relation with the second goal, a novel statistical approach based on re-weighted extended Procrustes analysis has been developed to georeference range dataset and to control its accuracy. Particularly, a high precision topographic network has been required in order to define a precise and stable reference frame.

Therefore, a novel flowline for health monitoring of complex and large dams which incorporates a new way of modelling real-world (scanned) objects has been developed. Regarding to the results obtained, the point-based computational techniques developed has enabled us to diagnose two main deformations: (i) an expected deformation due to the periodical processes produced when the dam is emptied or filled; (ii) an unexpected deformation due to the construction of a subterranean gallery. Finally, beyond the current scope of this paper and considering that the deformation analysis has been performed primarily in the direction normal to the surface of the dam, more deformation analyses could be applied in other directions. That is, testing whether the dam wall is sinking and even trying to analyze the correlation between these movements and the change of the surface in the normal direction.

## Figures and Tables

**Table 1.** Specifications of the TLS used in this research, features referred to vendors documents [22].

	**Efficiency**
Measurement range	200 m (optimal); 350 m (maximum)
Standard deviation:	1.4 mm £ 50 m; 2.5 mm at 100 m 3.6 mm at 150 m;
Single point accuracy:	6.5 mm at 200 m
Horizontal angular accuracy (deg):	12 mm at 100 m; 7 mm at 50 m
Vertical angular accuracy (deg):	0.0012
Circular level sensibility:	0.0014
Dual-axis compensator sensibility:	8′
Automatic leveling compensation	6
Spot size (beam diameter):	In real time
Spot size with autofocus:	3 mm at 50 m
Laser spot spacing:	0.3 mm at 5 m; 0.9 mm at 15 m; 1.5 mm at 25 m
	3.2 mm at 100 m

**System specifications**	

Type of laser:	532 nm. Time of flight. Green spot.
Class:	IEC 60825-1Clase 3R; 21 CFR-1041.10: Class 2
Field of view:	360° × 60°
Video camera	Digital image in real time with 5.5 × optic zoom
Beam deflection system	Oscillating mirror

**Table 2.** Details about the measurement periods.

1^st^ Period (May, 2006) Goals	2^nd^ Period (October, 2006) Goals	3^rd^ Periods (March, 2007) Goals
**Initial state-Dam almost empty**	**After six months-Dam** **completely full**	**After subterranean gallery** **construction** **Dam completely empty**
- Establish an initial state (null deformation) of the structure - Establish the reference coordinate system - Digital and metric archiving of the structure	- 1^st^ structural monitoring of the structure - Confirm and analyze its possible deformation	- 2^nd^ structural monitoring of the structure - Confirm and analyze its possible deformation. - Diagnose unusual deformations due to a subterranean gallery construction

**Table 3.** Reference block: standard deviations.

**Network adjustment** (4^th^ iteration)				
Period	Control Points	Standard Deviation (mm)		
		*σ_X_*	*σ_Y_*	*σ_Z_*
May 2006	I7	1.3	1.4	2.8
	I1	0.7	0.5	1.2
	I4	2.4	1.8	3.1
	I3	2.6	1.5	2.4
	PA1	1.7	1.2	2.6
	PA2	0.5	1.5	2.2
	PA3	2.3	1.2	3.2
	PA4	2.1	1.2	2.6
	PA5	1.7	1.9	2.1
	PA6	1.9	1.6	1.9

**Table 4.** Parameters for the re-Weighted Extended Orthogonal Procrustes (WEOP) transformation between the topographic and laserscanning coordinate systems.

Scale factor	Rotation (Grad)			Translation (m)			RMSE(m)
*c*	*ω*	*φ*	*k*	*tx*	*ty*	*tz*	RMSE
1.0001	0.0324	0.0406	200.9251	100.902	5.256	-0.216	0.003

**Table 5.** Parameters for the parametric 3D surfaces based on Radial Basis Functions (RBFs).

**3D surface parameters (1^st^ Period)**				**3D surface parameters (2^sd^ Period)**			
RBF centres	N° of points	N° of triangles	RMSE	RBF centres	N° of points	N° of triangles	RMSE

110296	4666051	8688803	0.003	119395	4984299	9023803	0.003

**Table 6.** Parameters for the parametric 3D surfaces based on Radial Basis Functions (RBFs).

3D surface parameters (2^sd^ Period)				3D surface parameters (3^rd^ Period)			
RBF centres	N° of points	N° of triangles	RMSE	RBF centres	N° of points	N° of triangles	RMSE

119395	4984299	9023803	0.003	123112	5236666	9147907	0.002

## Appendix A. Radial Basis Function (RBF)

RBF is a real-valued function whose value depends only on the distance from the origin, so that *φ(x)*=*φ(‖x‖)*; or alternatively on the distance from some other point *c*, called a centre, so that *φ(x,c)*=*φ(‖x-c‖)*. This function represents a signed “distance” from the object's surface and is an explicit function of position. Points inside the object have a negative “distance” while points outside are positive. The object's surface is defined implicitly as the zero set of this function.

Radial basis functions are typically used to build up function approximations of the form (11)

(11) $$y(x)=\sum_{i=1}^{N}w_{i}\varphi \left(\left\|x-c_{i}\right\|\right)$$

where the approximating function *y(x)* is represented as a sum of radial basis functions, each associated with a different centre *c_i_*, and weighted by an appropriate coefficient *w_i_*. Approximation schemes of this kind have been particularly used in time series prediction and control of nonlinear systems exhibiting sufficiently simple chaotic behaviour. RBF is popular for interpolating scattered data as the associated system of linear equations is guaranteed to be invertible under very mild conditions on the locations of the data points. Particularly, a surface modelling based on RBF represents the following advantages:

- It can be evaluated anywhere in 3D, on and off the surface, independent of the locations of theoriginal data points.
- Gradients and higher derivatives are determined analytically. They are continuous and smooth.
- The signed distance function fitted to the surface data forms a solid model.
- Interpolation and extrapolation are inherent in the functional representation. Consequently, thisapproach can be applied to the problem of mesh repair where incomplete meshes require holefilling and forming closed.
- An analytic model can be used to produce a smooth filleted join between two separate objects.
- An analytic model offers new approaches to the problems of mesh simplification, compression, morphing and reconstructing an object from scattered point cloud data.

## Appendix B. Weighted Extended Orthogonal Procrustes (WEOP)

The Procrustes analysis is a linear least-squares solution to compute the registration transformation parameters between two or more model points matrices up to their maximal agreement. Since its functional model is linear, it does not need initial approximations for the unknown transformation parameters. Though it was applied at first as a useful tool in factor analysis, it has become a popular method of shape analysis [10], and recently some authors have proposed the application of the method (with some variations, as generalized orthogonal Procrustes analysis or weighted Procrustes analysis) in geodesy, to transform 3D coordinates related to WGS 84 reference ellipsoid into 3D coordinates of a local reference system [4], and also in photogrammetry, to adjust blocks of photograms by independent models [5].

The method is able to directly estimate the translation vector **t**, the scale factor *c* and the orthogonal rotation matrix **R**, by which **A** is transformed to best fit **B**. This is achieved by searching the condition of minimum tr (**E^T^WE**)=min for the following function (12),

(12) $$\text{tr}\left[{\left(c\text{AR}+j{\text{t}}^{\text{T}}-\text{B}\right)}^{\text{T}}\text{W}\left(c\text{AR}+\text{j}{\text{t}}^{T}-\text{B}\right)\right]=\min$$

Solving this system the unknowns **R**, *c* and ***t*** are obtained (13),

(13) $$\begin{array}{ll} \text{R} & ={\text{VU}}^{\text{T}},\\ c & =\frac{\text{tr}[{\text{R}}^{\text{T}}{{\text{A}}_{\text{w}}}^{\text{T}}(\text{I}-({\text{j}}_{\text{w}}\;{{\text{j}}_{\text{w}}}^{\text{T}}/{\text{N}}_{\text{w}})){\text{B}}_{\text{w}}]}{\text{tr}[{{\text{A}}_{\text{w}}}^{\text{T}}(\text{I}-({\text{j}}_{\text{w}}\;{{\text{j}}_{\text{w}}}^{\text{T}}/{\text{N}}_{\text{w}})){\text{A}}_{\text{w}}]},\\ \text{t} & ={\left({\text{B}}_{\text{w}}-c{\text{A}}_{\text{w}}\text{R}\right)}^{\text{T}}\;{\text{j}}_{\text{w}}/{\text{N}}_{\text{w}}, \end{array}$$

where, **A_w_**=**W**·**A**, **B_w_**=**W**·**B**, and ***j*_w_**=**W**·***j***, and **V**, **U** are orthonormal eigenvector matrices obtained using Singular Value Decomposition (SVD) of matrix product (14),

(14) $$\text{svd}\left\{{{\text{A}}_{\text{w}}}^{\text{T}}\left(\text{I}-\frac{{\text{j}}_{\text{w}}\;{{\text{j}}_{\text{w}}}^{\text{T}}}{{\text{N}}_{\text{w}}}\right){\text{B}}_{\text{w}}\right\}={\text{VDU}}^{\text{T}}$$

being **D** the diagonal eigenvalue matrix.
